# Exploring the potentials and pitfalls of work experience and widening participation through narrative interviews

**DOI:** 10.1186/s12909-026-09308-2

**Published:** 2026-04-28

**Authors:** Alice Green, Dawn Jackson, Derek Ward

**Affiliations:** 1 College of Medicine and Health, University of Birmingham, Edgbaston, Birmingham, B15 2TT UK; 2 College of Medical and Health, University of Birmingham, Edgbaston, Birmingham, B15 2TT UK

**Keywords:** Undergraduate, Selection, Widening Participation

## Abstract

**Background:**

Applicant work experience (WE) is a non-academic criteria considered by many medical schools in their admissions processes. Concerns have been raised within the literature that access to some WE opportunities may be more elusive for students from less advantaged socioeconomic backgrounds (LA-SDBs). Few qualitative studies exist which explore applicant experiences of WE, and its contribution to the widening access agenda. Our qualitative research explores the WE of applicants from a variety of socio-demographic backgrounds, and draws on Bourdieu’s concepts of ‘capital’ and ‘habitus’ as tools which enable critical analysis of the role work experience may play in the reproduction of inequality.

**Methods:**

Narrative telephone interviews were undertaken with 23 applicants to a large United Kingdom medical school in the 2017–18 admissions cycle, purposively sampled backed on school background, age, gender and widening participation experience. Framework analysis was applied to interview data, and themes and subthemes identified.

**Results:**

WE remains a key step in the preparation process for applicants when applying to medical school. Participants suggested WE has the potential to confirm career choice and deepen understanding about the realities of a career in medicine. At times, applications from LA-SDB’s were provided with application support from healthcare professionals through WE which may have otherwise been inaccessible. Access to WE opportunities depended on access to information networks or school support, typically a barrier for those from LA-SDB’s, and myths and misinformation about WE were more commonly evident in this group.

**Conclusion:**

Meaningful and equitable engagement in WE requires a consideration of more than just access to WE opportunities. Our results suggest that applicants require timely and transparent information on how best to engage with WE opportunities. Additionally, communication with under-represented schools, directed at dispelling myths and fostering meaningful reflection, may go some way to mitigate the risk of social and cultural disadvantage in applicants from LA-SDBs.

**Supplementary Information:**

The online version contains supplementary material available at 10.1186/s12909-026-09308-2.

## Background

There is increasing momentum to enhance the diversity of medical school cohorts, to better reflect the heterogenous populations that healthcare serves, and to address longstanding inequalities affecting groups historically under-represented in medicine [1]^.^ Both investment and policy have directed widening access (WA) activities to increase the representation of applicants from less advantaged sociodemographic backgrounds (LA-SDBs) within medical schools, and ultimately to encourage social mobility and develop a diverse workforce of clinicians who are more likely to practice in underserved areas post graduation [1, 2]. Despite some progress through these initiatives, those from LA-SDBs remain underrepresented in medical school cohorts [3–5]. LA-SDBs’ refers to applicants who face structural disadvantage in the UK, including limited economic, social, or cultural capital and reduced agency over life opportunities. Indicators such as income, occupation, school type, educational attainment, gender, ethnicity, language, and special educational needs are commonly used to ‘measure’ disadvantage, but remain only proxies for a complex and multifaceted concept [6].

The heterogeneity and complexity of the applicant experience makes it difficult to articulate a single solution to turn the tide of disadvantage in medical school admissions. However, the consensus within the literature suggests that successful and sustainable change will require more than just alterations to selection processes, or the introduction of widening access schemes [5, 7, 8].

In this study, we have explored the preparatory experience of applicants, with a particular focus on work experience (WE) as a window to the socio-cultural context in medical school selection.

The landscape of the medical application process has changed from one which considers solely academic criteria, to one which incorporates non-academic criteria in the assessment of candidates [9, 10]. WE is one such non-academic criteria considered by many medical schools, offering a means for applicants to demonstrate an appreciation of the demands of a career in medicine, alongside the personal attributes desirable for a career in this field [11–14].

Definitions of the types of WE taken into account by medical schools vary from institution to institution. In the broadest sense WE pertains to an activity in a health-related or public-facing workplace, and can involve observation, volunteering or paid employment. Medical schools are typically not prescriptive on the type or length of experience needed [11, 15, 16]. However, it is unclear whether this flexibility is reflected uniformly in the implementation of admissions processes, particularly as selectors have historically favoured clinical-based placements in their decision-making [12, 17].

The evidence suggests that the vast majority of UK medicine applicants complete WE in some form, highlighting its significance to applicants [18, 19]. However, concerns have been raised within the literature regarding the accessibility of WE opportunities, some of which may be more elusive for students from lower socioeconomic backgrounds [12, 20–22]. Few qualitative studies exist which explore applicant experiences of WE, and the contribution of WE to the widening access agenda is sparely investigated. It has been suggested that applicants may perceive WE as a ‘tick box exercise’ to getting into medical school, rather than as a useful experience to gain deeper insight into a career in medicine [23]. WE can be perceived by applicants as an exercise in ‘who you know’ [24], and barriers to accessing opportunities are outlined elsewhere in the literature [20–22, 25].

From the widening access perspective, there is little in-depth data on how these barriers manifest and how extrinsic influences, such as social networks and institutions, may act to aid, or hinder, the applicant [18, 24, 26–31]. If WE is perceived as an exercise in social currency, it may follow that it acts as a deterrent for low income students even before they apply [20].

### Conceptual framework

These studies begin to pull at an intricate thread of complexity and disadvantage in medicine admissions. However, efforts by university admissions teams to support the opportunities afforded to applicants may neglect the nuanced effects of socialisation that lead to the ‘know how’ required to succeed.

Bourdieu’s capital-field-habitus framework is a useful lens through which to consider individual experiences within the socio-cultural context, attending to the inequalities that can be reinforced in the field of education [32]. Bourdieu suggests that successful students learn the ‘rules of the game’ in the field; how to act, how to meet expectations and how to engage with the community. Others are less adept at navigating these rules or positions, and therefore remain less successful [33].

Bourdieu’s concept of ‘habitus’ suggests that inequalities are set in motion long before an applicant engages with WE. Habitus represents the legacies of family and childhood socialisation that have brought about a set of complex predispositions, which incline applicants to act or react in certain ways, and which generate perceptions, attitudes and practices [33, 34]. The concept of habitus is frequently cited in the literature examining access to higher education [35–37], where such predispositions can render students from outside the predominant culture unable to decode the implicit “rules of the game” [35, 38, 39]. Habitus has been considered in research relating to ‘first in family’ students, and ‘working class’ medical students, where distinct disadvantages were suggested in basic knowledge about secondary education, family income and support, education expectations, academic preparation [36] and insufficient school support [28].

Bourdieu considers four types of capital: economic, cultural, social and symbolic. Each represents an area of potential advantage to be leveraged by an agent to get ahead. Economic capital in our setting may represent an applicant’s access to funds to travel for WE, whilst social capital may refer to networks able to provide advice, or help to secure a WE placement. Cultural capital may be gained by being proficient in the language or practises of a particular culture, such as healthcare [40]. Brosnan’s work explores how Bourdieu’s concepts illuminate the structural and cultural forces shaping medical career trajectories [41]. Applying this lens earlier in the pathway is therefore well suited to our focus, as work experience is applicants’ first point of entry into the medical field, where inequalities in capital and habitus can begin to influence. Within the literature, studies related to medicine admissions frequently explore application preparation for medical school selection through the quantification of activity, and association with success [42, 43]. However, the literature is less clear on how WE is negotiated and perceived by applicants in practice, and how these experiences vary between socio-demographic groups. It is imperative that we understand the ways in which applicants understand, negotiate and navigate their own ideas of WE requirements in medicine selection. Our qualitative research, drawing on applicant narratives, explores the WE of applicants from a variety of socio-demographic backgrounds, and draws on Bourdieu’s concepts of ‘capital’ and ‘habitus’ as tools which enable critical analysis of the role work experience may play in the reproduction of inequality [40, 44].

## Methods

### Research aims

1. To explore applicants’ experiences and perceptions of WE.
2. To investigate the facilitators and barriers faced by applicants when accessing WE.
3. To explore the value of WE for applicants.

This study takes a social constructivist approach, conceptualising shared knowledge constructed through social interaction and acknowledging the roles of researcher and participant in its co-construction [45]. In this paradigm, the aim is to understand particular situations, drawing on the individual perspectives of participants, and emphasizing the importance of interpretations, culture and environment [46]. The premise of narrative enquiry is that, as humans, we come to understand our lives, and give meaning to our lives, through story [47]. Narratives offer insights to the wider socio-cultural context, illuminating the narrator’s past, present and imagined future, as they attribute meaning to their experience [48].

### Interviews

Narrative interviews with medical school applicants were conducted during November 2017 until February 2018, a window in the UK admissions calendar when applicants have submitted applications, but typically have not received offer decisions. Telephone interviews were chosen to facilitate participation across the geographical breadth of the UK, and to encourage discussion of sensitive information, without the pressure of face-to-face contact [12]. Interviewers aimed to build rapport with respondents and offered advance information about the interview focus, to facilitate discussion [18, 19, 23]. Opening statements and approach to the narratives were developed in a workshop for all interviewers (DJ, GS, NW, and HW) to ensure consistency in interviewing. The interview schedule is shown in Appendix 1 (Interview Schedule).

The interviews typically lasted 30–50 min. Table 1 outlines respondent characteristics, from a variety of school and demographic backgrounds [24], We aimed to recruit around 20 participants, to provide sufficient insight to experiences across a variety of socio-demographic background [31]. Each interview was audio-recorded and transcribed verbatim by the interviewer shortly afterward.

**Table 1:** Participant demographic data and outcomes at selection

Participant number	School	Ethnicity	Contextual data used to offer interview	Interview	Offer	Contextual data used to give offer
1	SNS	White British		Y	Y	
2	SNS	White British	Y	Y	Y	
3	SNS	White British		Y	Y	
4	SNS		Y	Y	Y	
5	SNS	White British	Y- but below threshold	N	N	
6	SNS	White British		Y	Y	
7	SNS	White British		Y		Y
8	SNS, WP	Asian Pakistani	Y	Y		Y
9	SNS	Asian other		Y	Y	
10	SNS	Black Caribbean		Y	Y	
11	SS	White British		Y	Y	
12	SS	White British		N	N	
13	SS	Mixed White/Asian		Y	Y	
14	SS	Asian Indian		Y	Y	
15	SS	Asian Pakistani		Y	Y	
16	SS	Black Caribbean	Y	Y	Y	
17	Ind	White British		Y	Y	
18	Ind	Asian Indian		N	N	
19	Ind	White British		Y	Y	
20	Ind	White British		Y	Y	
21	Ind	White British		Y	Y	
22	Ind	Asian Indian		Y	Y	
23	Ind	Black African		N	N	

Key:

SNS = State Non-Selective

SS = State Selective

Ind = Independent

WP = Widening Participation

NB: all participants provided consent to crosslink their demographic information (provided on online survey) and outcomes at selection

### Identifying participants

The University of Birmingham in the United Kingdom receives over 2000 applicants annually for around 380 undergraduate places [49]. It has embedded a series of processes and support systems aiming to reduce inequalities for applicants from LA-SDB’s, including the use of “contextual data” to reduce selection requirements and providing widening access programmes for additional support with applications [50].

In the 2017/18 application cycle, all ‘home’ applicants to the University of Birmingham standard 5-year MBChB (medicine) degree were invited to complete an online survey to register their interest, provide baseline demographic data and complete contact information to enable purposive sampling.

Applicants to medical school typically attend state nonselective schools (SNS), state selective schools (SS), or independent (private) schools (IND) [29]. In the UK, participation in IND or SS school education is linked with higher rates of acceptance to highly-selective universities [10, 28, 30]. However, children from low-income families (such as children who are eligible for free school meals) are least likely to attend IND or SS schools. At the time of recruitment to this study, just 3% of SS pupils were eligible for free school meals, despite a figure of 13% eligibility in the total UK pupil population [51]. Private education at IND schools (requiring fee-payments) is concentrated at the very top of family income distribution.^)^ School background was therefore chosen as one of the sampling criteria for the study, offering a rudimentary marker for SDBs in the UK setting [29]. Purposive sampling also considered gender and ethnicity.

For the purpose of this study, work experience was conceptualised broadly and included both clinical observation and activities involving caring or service roles. The Medical Schools Council guidance defines work experience as "any activity or experience that helps you prepare for medical school and develop skills", including developing "a realistic understanding of medicine, commitment and motivation, along with development of communication skills [52]. Within this study, voluntary activities were therefore included under the umbrella of work experience, as important insights would be missed if voluntary, non-paid activities were excluded.

### Data analysis

The 23 transcripts were analysed using framework analysis, as described by Gale et al. [53–55]. Framework analysis sits under the umbrella of thematic analysis [56, 57] and both focus on identifying relationships and differences within qualitative data, seeking to explore and understand [53]. Framework analysis emphasises how both prior ideas and emergent data driven themes should guide development of an analytic framework, a combination of which has been undertaken in this study [56].

**Fig. 1:**
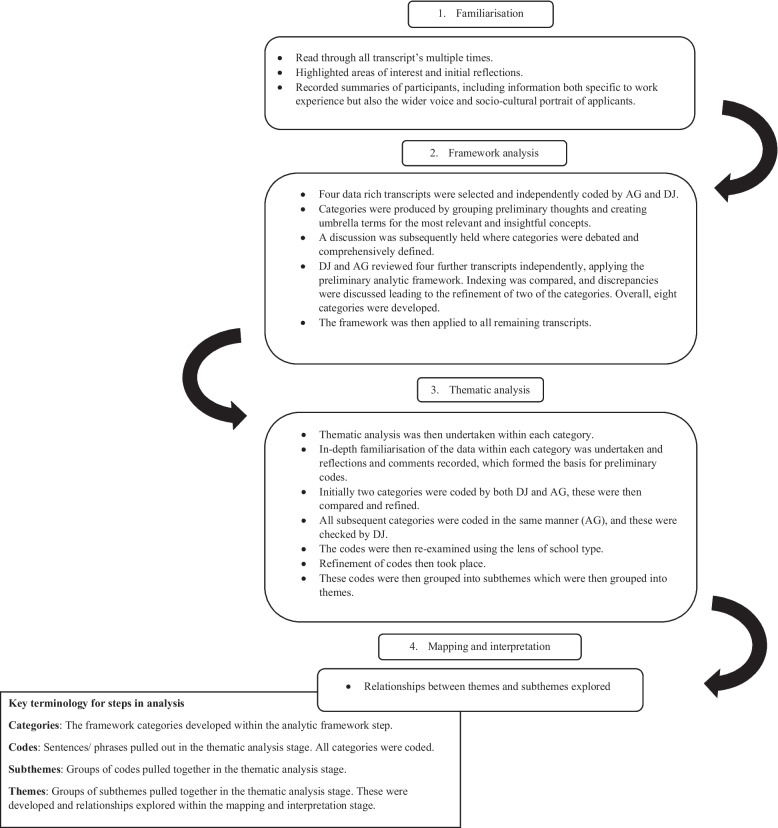
Framework analysis

The steps undertaken during analysis have been outlined in Fig. 1.

The primary researcher (AG) was blind to applicant outcomes at selection, to reduce any affect this had on interpretation of participants statements. Thematic analysis was undertaken using NVivo (QSR Version 12) [58].

## Results

Data saturation [59] was reached after 19 transcripts and no new codes were generated after this.

### Sample characteristics

The characteristics of the participants are displayed in Table 1.

### Themes

The data has been presented in two broad areas: *access to work experience* and *applicants’ opinions of work experience.* Themes and subthemes are summarised in Table 2. Bold demonstrates researchers own emphasis in quotations.

**Table 2:** Themes and subthemes

	**Theme**	**Subtheme**
Access to work experience	Facilitators	Work experience programmes
		Social networks
		School types
		Peer groups
	Barriers	Information barriers
		Late applications
		Logistical barriers
Applicants’ opinions of work experience	Perceptions	Aid career choice
		Quota fulfilment
	Reflections	Confirm career choice
		Shifting attitudes
		Variable quality
	Clinical shadowing versus volunteering	
	Variability and applicants’ insight into fairness	

Supplementary quotes are detailed in Appendix 2. The synthesis and interpretation of these results has been presented within the discussion.

#### Facilitators and barriers to accessing work experience

##### Facilitators to accessing work experience

**Work experience programmes**

Some hospitals offered formalised WE programmes accessed via applications. Applicants felt these improved ease of access to WE opportunities, and some felt the structured nature of such programmes offered better quality experiences.

[Work experience] was **extremely beneficial because it was, you know, properly organised.** – Participant 17 SS.

**Social networks**

Most applicants used social networks to secure at least one WE opportunity, and some suggested that minimal effort was required on their part due to these contacts:

It was**, just family connection** that got me that. I’m not sure how, you know, the inner workings of it were, because **I just got a message** saying you know, you can come down for these two days – Participant 17 IND.

Having the right contacts was seen by some applicants as uniquely important for securing hospital-based experience.

**Getting any kind of hospital work experience is so difficult unless you actually know someone**. – Participant 15 SS.

Although most opportunities came from informal social contacts, some students, most often from SNS schools, obtained WE through contacts from personal health conditions, whereas students from IND schools exclusively utilised ‘family friend’ networks. SNS students were more likely to report social networks or perceived lack thereof as a barrier to WE. This variation in access seemed to be well-recognised and students reflected on disadvantages this may have.

I quickly realised that I was not in the same situation that some of my classmates were in, that they had an uncle in the NHS, so they could just go and do work experience with them – Participant 1 SNS.

In addition to providing access to WE opportunities, social networks were also perceived to provide access to ‘insider information’ about steps within the application process, increasing applicants’ awareness about requirements.

**School support**

IND schools were typically more proactive in their approach, disseminating information earlier and encouraging students to get WE sorted early.

**Almost as soon as I’d expressed my interest**…we started having a few talks with the teacher about you know, what, what do you need to do to start preparing. – Participant 17 IND.

Constraints on school support offered were discussed more frequently from applicants attending SNS schools, particularly relating to challenges in obtaining sufficient and timely information regarding applications.

**Peer support**

Applicants reflected that peer support helped through the sharing of information or opportunities, and also helped them to feel less isolated.

I think there was also encouragement in the fact that all my volunteering was, you know, other people had done it before… this idea of doing things together was good encouragement. – Participant 13 SS.

### Barriers to accessing work experience

#### Information barriers

The applicants’ accounts suggested that access to information about WE opportunities was unequal and often mediated by institutional support. Applicants with access to school guidance or established social or family networks were more likely to identify opportunities early, whereas others described a more fragmented and time-intensive process when attempting to navigate available placements independently, sometimes yielding little success:

It was really difficult to find work experience. It’s not really a website, everyone keeps saying look at different adverts, look at university hospitals – Participant 18 IND.

Applicants with additional support from schools or social networks typically encountered less information barriers when researching opportunities.

They [the school] were really good at getting us to do it quickly and then if you couldn’t find any, they’d send us, like ‘ok, there’s this – Participant 2 SNS.

#### Late applications

A number of participants discussed how the timing of information and institutional guidance influenced access to WE opportunities. Applicants who became aware of opportunities later in the application process described missing deadlines or encountering oversubscribed programmes, suggesting that knowledge of *when* to apply formed an additional layer of advantage within the preparation process.

Several applicants referred to the importance of starting ‘early’ to arrange WE, some as early as aged 14–15. High competition for limited WE places appeared to contribute to this drive. Many students, particularly those from SNS schools, expressed instances of being unable to access opportunities due to missing application deadlines or oversubscribed programmes. Support from schools seemed to mediate this, through the provision of timely information, or by arranging placements on behalf of the applicant.

That’s one of the things that I think could maybe be a disadvantage to people who want to apply to medicine …

… **if they don’t have access to that information, it’s kind of like, how would you know, or they might think “oh I’ve got till January to apply” and then like oh it’s actually too late I’ve missed the deadline.** – Participant 4 SNS.

#### Logistical barriers

Some applicants were not eligible to participate in local hospital programmes, as they lived outside the geographical boundary for eligibility. Many discussed problems with communication from placements, receiving delayed replies to their application, or none at all.

they didn’t get back to me so I emailed again and they said my email was lost. So I emailed again and they didn’t reply for like weeks and weeks… That was really xxxx. In the end they said that they didn’t have any work experience left. – Participant 11 SS.

Perceived legal barriers related to the applicant age (and associated insurance concerns) were frequently cited by placement providers, leading to further difficulties for some applicants. Legal barriers were especially associated with clinical shadowing, although school support and family connections helped to mediate this.

I wasn’t able to find anywhere for work experience myself because I have a late birthday so I wasn’t 16 yet, which caused me problems… but the school has a link with the women’s hospital, which enabled me to get a 10-day work experience place – Participant 13 SS.

##### Applicants’ views on work experience

Applicants shared their perceptions prior to undertaking WE and also reflected on their experiences after completion.

### Perceptions of work experience

#### Aid in career choice

One of the main motivators to undertake WE was to gain a realistic understanding about medicine as career.

I kind of wanted to see if that was the idea of it or if it was the terrible thing that the news seems to make it out to be, long hours, and kind of find out what the reality was behind it. – Participant 19 IND.

#### Quotas to fulfil

Some applicants believed accumulating as much WE as possible was of overall importance. Generally, there was a sense that two weeks WE was perceived to be the optimum amount, with one student taking time off school to fulfil this perceived requirement.

I took a week off because **I knew it was important that I had two weeks.** – Participant 11 SS.

Some applicants valued a variety of experience, such as WE in various hospital departments or nursing home settings.

I’ve had a full range of experiences**, to make my application really stand out** and have more stories to tell. – Participant 18 IND.

For some applicants, these perceptions increased stress and anxiety about the application process when they were unable to fulfil them.

A few people told me that… it was like a compulsory thing so I was really stressed because all the GPs that I contacted in the area said that they couldn’t because of insurance so like I was despairing like, “oh no **I couldn’t get into Medical School because of this”**. – Participant 11 SS.

Some WE opportunities were more coveted than others, and many suggested that volunteering in nursing homes were the ideal WE placements.

I tried old people’s homes for volunteering…that’s the example most medical, most successful medical applicants give. So I looked there but in my area they were already full up. – Participant 16 SS WP.

### Applicants’ reflections on work experience

#### Confirm career choice

Many applicants enjoyed their WE, and felt that it helped confirm their career choice. Some had been inspired to apply to medicine because of WE, typically through encountering positive role models.

I shadowed the neurosurgeon which I found just so amazing. And think, from that point, I’ve always wanted to do it. – Participant 5 SNS.

Some applicants felt that WE provided a unique insight to the realities of the role of a doctor, both positives and negatives, helping them make informed decisions when applying.

I think you don’t really have that good of an idea until you’re actually in that environment. – Participant 22 IND.

#### Shifting Attitudes

WE was also perceived to have developed wider views around healthcare, and contribute to an evolution of previously held views, towards greater alignment with those of clinical professionals.

I looked at like old people used and not in a, not in a spiteful way but thinking how much keeping the older generation alive sucks money out of the NHS [UK National Health Service]. And then **when I was actually on the ward and I saw these people as individuals** and that they need healthcare **it really made me stop and think and re-evaluate** how I think about money and resources. – Participant 16 SS WP.

Some applicants’, including those from SNS backgrounds, reported that information gained from WE impacted and changed their approach to their application.

He’s [a consultant on work experience] the one who advised me that I should be spending time in a care home …

…So, as soon as I got back from that placement, I applied to care homes – Participant 9 SNS.

WE seemed to act as a springboard for other opportunities. Placements facilitated applicants to make professional contacts, who opened up additional opportunities, and this was particularly valued by those from SNS schools.

I was able to email these people who have positions in the training sector of the hospital… I really wanted to be able to do this two days of A&E that I’d, I’d heard about, I was able to contact them. – Participant 6 SNS.

#### Variable quality

Not every placement was perceived to be positive one. For Participant 9 (SNS), their initial WE fell short of their expectations so they felt driven to organise subsequent placements to meet these desires. Another applicant suggested that instead of being inspired and motivated by role models on their WE, they had felt lost:

I had two days shadowing a cardiac consultant but he didn’t really expect me. When I turned up he was like “oh what are you doing here you’re supposed to be here in two weeks” and I was like “no, this is the day” and so I did feel really out of my depth. –Participant 4 SNS.

For some applicants WE seemed to be perceived as an exercise important to complete, but with limited deeper reflection expressed on its potential benefits or purpose.

I started it with the **intention of knowing it was important for applying to medical school** and I just kept going with it. – Participant 1 SNS.

#### Volunteering versus clinical shadowing

Interviewees appeared to perceive a divide between volunteering and clinical shadowing, Volunteering was perceived to be more socially orientated and linked with the development of skills.

I thought also that more volunteering is more about, more shows you’re caring. I feel like with work experience, even if I didn’t have any work experience which I did, it wouldn’t really change my application all that much. I thought that **volunteering really shows that I’ve done something**. – Participant 18 IND.

Applicants describing clinical shadowing, were more likely to use passive language such as ‘saw’ and ‘observed.’

In terms of what I did, it was most just you know, just **sitting** in. Or you know**, walking round**, **observing** all the different things. – Participant 15 SS.

Applicants’ depth and quality of reflection also appeared to be enhanced when describing volunteering placements. For example, Participant 8’s account of clinical shadowing was ‘list-like’ and descriptive, in comparison to their rich reflection on volunteering activities and the skills they acquired.

During the experiences you kind of get that, kind of get an **understanding and experience** of **how to talk** to a lot of different, a lot of variety of people and how you can compose yourself. You have to be patient with some people, you have to be a lot clearer with some people than others and the different ways that you **communicate** with different people, so it’s really given me experience about that. – Participant 8 SNS WP.

### Applicants’ insight into variation and fairness

Due to unequal and varying access to WE, facilitators for some students manifested as barriers for others.

Applicants who appeared disadvantaged in the extent of school support received, or access to social networks, expressed limited insight into their own disadvantage.

A minority of applicants did appear to recognise the issue of ‘fairness’ in securing WE opportunities, particularly with respect to accessing information and networks:

Because it’s such a, kind of isolated process in that sense it means people who have had a lot of experience, they have been looking at it in advance, they have a better chance. **It doesn’t necessarily mean they’re better candidates it just means they’re more aware of all these hurdles that they have to overcome.** So I think to make it a more even playing field everyone should have a good understanding of what each thing is, what they need. – Participant 4 SNS.

## Discussion

Overall, WE remains a key step in the preparation process for applicants when applying to medical school. Our participants suggested WE is a means to confirm career choice, enhance knowledge and deepen understanding about the realities of a career in medicine. Applicants reflected on the value of learning from positive role models, and WE challenged attitudes towards health and equality, providing an instrument aiding applicants’ introspection. From a widening participation perspective, WE may also ‘level the playing field’ for some applicants, providing access to insight and support from professionals about the application process, which may have otherwise been inaccessible.

However, the data also highlights the variability in applicant experience, with respect to accessibility of opportunities, alongside myths and perceptions surrounding the process.

Drawing on Bourdieu’s concepts of capital and habitus, our results suggest that access to WE often depended on access to informal social networks or school support. Formal WE programmes also had the potential to be a barrier for some applicants due to geographical constraints, oversubscription, legalities and logistical timings of applications. In addition, navigating information on WE opportunities was time-consuming and stressful for some applicants, whilst for others, opportunities were facilitated with minimum personal effort due to extrinsic support. Applicants from SNS schools and those considering medicine later in their school career appeared to be particularly disadvantaged in this regard.

Insufficient school support has been linked with ‘habitus’ when considering disadvantage in the LA-SDBs medical applicant experience, and our results corroborated this [28]. Those from IND and SS schools typically recounted proactive school support, with dedicated time and resources provided to help secure WE. In contrast, this was less apparent in the stories of applicants from SNS schools, where school support more frequently seemed reactive and involved signposting applicants, rather than active provision of resource.

Associated closely to habitus, social capital, through the leveraging of healthcare connections in social networks to secure WE, appeared less available to participants from SNS schools and seemed to be a recognised barrier to obtaining experience [24, 28, 60].

Bourdieu’s concept of ‘cultural capital’ relates to the students’ ability to decode the ‘rules of the game’ in the field of WE, enabling successful navigation of opportunities [32, 33]. Those from SNS schools appeared particularly susceptible to myths and assumed ‘truths’ surrounding the merits of particular types, durations and breadths of WE. This, at times, led to additional effort securing placements, and subsequent reflections that these efforts may not have yielded proportionate results. These applicants were more likely to describe feeling ‘lost’ during WE placements, struggling to orientate or engage in the healthcare environment. In addition, we also noted that many of the SNS applicants appeared to lack insight into the extent of their disadvantage, and we felt they risked overestimating the quality of support they had received.

We observed variable depth and quality of reflection on WE across the applicant stories, regardless of school background. While some applicants richly reflected on learning gained, others appeared to see placements as a ‘tick box exercise’, with the acquisition of placements as the overarching goal. The applicant stories suggested that volunteering may offer a different experience to clinical shadowing, and potentially one in which applicants reflected more deeply on their experience, attitudes and learning. This reflects a broader tension in the role of WE within the application process. While some applicants appeared to approach placements as a requirement to accumulate experiences or fulfil perceived expectations, others described intrinsic benefits such as confirming career choice, gaining insight into the realities of medical practice, and learning from professional role models. These findings highlight the importance of not only improving access to WE opportunities, but also supporting all applicants to engage meaningfully with these experiences and reflect on their relevance to a future career in medicine.

Almost no participants reflected on strategies, approaches or resources used to help them engage meaningfully with these experiences. Whilst this may reflect the nature of the study setting, it raises questions about the extent to which applicants are prepared to understand and engage with the purpose of WE, in the intended spirit of realistic career exploration and personal reflection. Cultural capital is closely linked to upbringing and habitus [61] and, as suggested in the variability of our applicant reflections, students will vary on the support they will require to feel able to navigate and meaningfully engage in a healthcare environment [24, 61].

### Recommendations

Our results suggest that WE does have the potential to confirm career choice for applicants, and to deepen their understanding of themselves and the healthcare environment. From a widening participation perspective, the contacts and support gained from a WE placement may go some way to rectify unequal access to social and cultural capital in the application pathway.

At face value therefore, WE arguably has the potential to enrich the applicant journey, and level the playing field in medicine admissions.

However, variable logistical and geographical implementation of WE, coupled with considerations of student habitus and capital, indicates a need for caution and appreciation of the equity of WE by admissions teams. The advent of virtual WE, accelerated by the Covid-19 pandemic, offers one means to standardise and improve accessibility to WE opportunities [62, 63]. These resources embed tools to help applicants meaningfully reflect and engage with the material. However, when we consider the complexity linked to the longstanding predispositions of capital and habitus, it is unclear if these sources of support would be sufficient for all. Intuition would suggest that tailored support for applicants would be ideal, but in the resource-constrained settings of SNS schools and overwhelmed institutions, this may serve to perpetuate the inequalities in access that currently exist. Pragmatically, we would suggest that all medical schools should make applicants aware of virtual opportunities for WE, and also provide information and tools (such as reflective diaries) to assist applicants to meaningfully engage with these platforms. When, and where, such information is disseminated also requires careful attention. For students who opt to pursue medicine later in their school career, or for those less ‘in tune’ with mainstream resources for information, there is a risk of ‘missing the boat’ for WE, or being ill-prepared for application. Timely communication directly with schools and careers advisors may go some way to mediate this.

Applicants appeared to view volunteering experiences as offering a richer experience, when compared to clinical shadowing. These reflections appear at odds with our survey study of applicant preparatory activities, where multivariate analysis indicated that clinical shadowing, and not volunteering, was significantly associated with success in securing a medical school place [19]. Granular study of the nature of WE, and exploration on the impact of the grade and types of healthcare professional who deliver it, is infrequently explored in the literature. Further research is required in this regard to better direct applicants to opportunities most likely to yield meaningful experiences.

### Strengths and limitations

Our data were collected before the Covid-19 pandemic, when many medical schools relaxed their requirements for work experience [15]. However, on review of 2023/24 admissions criteria, a number of UK schools have again outlined specific requirements for WE as part of their selection criteria, such as mandatory or ‘desirable’ experience in healthcare settings, discussion of work experiences within interview, and collation of ‘evidence’ of WE [14]. Our results therefore remain pertinent to current WE expectations.

A number of steps were taken to reduce perceived power imbalance in the interview process and to facilitate applicant narratives, by ensuring interviewers were not involved in selection processes, and care taken to build rapport in the interview process [48].

The study explores the experiences of both successful and unsuccessful applicants to a single UK medical school. However, further perspectives may have been identified by inviting participants from across the UK at an earlier stage in their school studies, as those who were unable to secure WE may have been deterred from applying to medical school altogether.

## Conclusion

The complexity and variability of applicants’ experiences make it difficult to propose simple solutions to entrenched disadvantage in medical school admissions. As medical schools and academic institutions continue to engage with the broader aspiration of equity, diversity and inclusion (EDI), this study serves as a call to closely examine the multiple facets of admissions processes, such as work experience, and to interrogate how these practices may reproduce or obscure underlying inequalities.

Although WE remains a cornerstone for many UK medical schools admissions processes, questions have been raised about its role within the widening access agenda. This study has identified benefits associated with work experience to enhance understanding and opportunities for some applicants from LA-SDB’s. However, unequal experience of facilitators such as social networks and school type, contribute to unequal access to WE opportunities, especially for students from SNS schools. Transparent communication from medical schools is therefore vital if we are to take steps towards mitigating the effects of ‘insider information’, and reduce myths and misinformation around WE. Medical schools must consider both the timeliness of this information, and its audience. Our research suggests that information about WE processes, purpose and engagement should happen earlier in the selection journey; with both applicants directly and the staff from under-represented schools who support them. Acknowledging the complexity of access to WE, alongside taking concrete steps to bridge the link between underrepresented secondary education providers and their local institutions, may shift the culture of access and aptitude being so closely interlinked and ultimately lead to increased diversity in medical school cohorts. The application of work experience within the UK admissions process must evolve, as currently we risk, in part, selecting students on the basis of social advantage.

## Supplementary Information

Supplementary Material 1.

## Data Availability

The datasets generated and/or analysed during the current study are not publicly available due to the risk that individual privacy may be compromised but are available from the corresponding author on reasonable request. Data supporting the results of this study have been provided in Appendix 2.

## Abbreviations

LA-SDB: Less advantaged socio-demographic background
UK: United Kingdom
WE: Work Experience
WA: Widening Access
SS: State Selective (school)
SNS: State non-Selective (school)
IND: Independent (school)
WP: Widening Participation
