# Supplementary material for: Exploring the potentials and pitfalls of work experience and widening participation through narrative interviews
Source: BMC Med Educ. 2026 Apr 28;26:959. doi: 10.1186/s12909-026-09308-2 (PMC13262470; doi:10.1186/s12909-026-09308-2)
Supplement: Supplementary file 1 — Supplementary Material 1. [file 12909_2026_9308_MOESM1_ESM.docx]

**Appendices**

Appendix 1: Interview schedule

Appendix 2: supplementary quotes

**Appendix 1: Interview schedule**

INTERVIEW SCHEDULE

***Introduction***

Thank you for agreeing to take part in this interview.

*Interviewer introduces themselves: name, background. Bit about their experiences of medical school application.*

The study aims to explore how you have prepared to apply for medical school.

You’ve been asked to take part because you are currently going through it, and therefore have up to date knowledge about what’s involved.

This interview hopes to explore your experiences.

The interview should last around 30 minutes.

You are free to decline to answer any question without reason.

You are free to withdraw from the study up to 28 days after the interview has taken place.

Please do let me know if you feel uncomfortable in the interview or wish me to pause.

Your name or any other identifying information will be removed so that your responses are anonymous when we write up the research for publication.

QUESTION:

So today’s interview, as I said, is about your experiences of preparing to apply for medical school.

Take time to think where you need to, you don’t have to launch in straight away

This is a different type of interviewing to maybe what you’ve you’re expecting. I don’t have a pre-defined list of questions to ask

I’m more interested in hearing your story and what you feel is important

So you won’t hear me speaking much

Don’t feel uncomfortable about that- that’s supposed to be the case. You might not hear much from me. But you can stop at any point and ask questions wherever you like.

**Can you tell me the story of your how you’ve prepared for applying to medical school?**

**You can start however you like, and think about the experiences, events or people that have helped, or hindered, and that you think are important to share.**

***PROMPTS (if needed)***

**Formal preparation** : UK CAT, interviews

**Informal preparation:**

Work experience

Voluntary experience

Paid work

Discussions

Reading

Hobbies

**Challenges faced**

# **Appendix 2 Supplementary quotes**

| Theme | Subtheme | Illuminating quote |
| --- | --- | --- |
| Facilitators | Work experience programmes | *I arranged work experience which took ages* ***like so many places were full, obviously it’s the same story for a lot of prospective students*** *who want to do medicine. – Participant 4 SNS*  *I went on a program that was organised by *[local] hospitals where they allowed me to go into surgery which I don’t think I would have been able to externally as I think you have to be over 18 to go. – Participant 22 IND* |
|  | Social networks | *For the other two they were through family friends and contacts that I had* ***so I managed to do a bit more work experience at those two, maybe a week at each, in a wide variety of different specialties and things. –*** *Participant 3 SNS*  *I have family friends, my sister goes *[to the university], she’s a medic, so I got a lot of information from her about the application process, and* ***I’d learnt from what she did*** *– Participant 22 IND*  *The connections, um, for example he was talking about how* ***he didn’t apply for work experience, his dad knew all these different doctors obviously and he just got him a place in that. I think that saves a lot of stress. –*** *Participant 16* *SS WP* |
|  | School types | *Partially, it was the all, you know****, obligation*** *of it all in a way. – Participant 13 SS*  *So they were really* ***good at getting us to do it quickly*** *and then if you couldn’t find any,* ***they’d send us,*** *like ‘ok, there’s this and you gotta pay this amount of money to do it’ – Participant 2 SNS*  *Every Friday, in the afternoon after lunch we did not have any scheduled lessons, but instead people who were applying to medicine or veterinary, there was a teacher at school who would talk to us about how applications were going. – Participant 21 IND* |
|  | Peer groups | ***It’s quite nice knowing that other people are going through it, kind of reassuring.*** *– Participant 23 IND*  *Talking to other medical applicants is helpful. Because it gives you an idea of not just what you’re going through now, not just the pressure, how you’re applying* ***but how other people are doing it, how they’re taking their approach to it and really give you like, a sense of, not of if you’re doing right or wrong but where you are and how to direct yourself and that direction*** *– Participant 8* *SNS WP* |
| Barriers | Information barriers | *If I hadn’t been to that day of them explaining everything,* ***I wouldn’t have known that****. So, I think there should be kind* ***of more understanding about the process of applying to medicine. –*** *Participant 4* *SNS*  *I did find that I’d spend a lot of time on my computer just reading****. Cos there are so many things accessible to me as well.*** *Especially certain websites that are designed for medical students, or mainly medical students. And I think that really* ***laid out things for me in a clear way****. And that suited me as well. So, yeah,* ***I spent a lot of my time researching. –*** *Participant 9 SNS* |
|  | Late applications | ***They said to us, they said if you’re doing medicine, do it now, like September, October, you need, you should have your placements sorted by the start of November at the latest****. So, I had my, I had mine sorted mid-October and I was ready to go for the next, you know the following June/July whenever I did it. – Participant 2 SNS*  *So, I didn’t manage to get any hospital work experience because my local hospital ran it on a first come, first served basis and it wasn’t very clear on their website and err,* ***I missed the cut off very very closely. Umm, so I didn’t get any hospital. –*** *Participant 17 IND* |
|  | Logistical barriers | *Oh, they only looked at like their kind, the area they’re in, *[that] area. And* ***I don’t really live in that area.*** *So, they kept on emailing with that, you contact a hospital in your area then you can try that- Participant 18 IND*  *After emailing round lots of GPs* ***none of them said that they would be able to because of insurance because I wasn’t 18*** *– Participant 11 SS* |
| Perceptions of work experience | Aid in career choice | *I still didn’t really have a clue as to what I wanted to do as a career, but that sort of made me decide when we got on to work experience later on* ***that I would try out hospital work experience to see if that would suit me****. – Participant 13 SS*    *Err, well, I guess the college and my, my family they said just go do work experience and see what working as, working as a doctor and the doctors’ role would be. Umm, really, well from the media you just get the doctor, obviously helping patients but* ***what do they do on a daily basis, but what do they do on a daily basis and how it’s structured, how the day is structured, what time do they go and what time do they come home****. – Participant 8 SNS WP* |
|  | Quotas | *I have done mainly hospital work experience and I have done half a day in a GP surgery, but the* ***remaining 9 days of my work experience*** *have been in various hospitals. I’ve* ***done paediatrics work experience, surgical work experience with a gynaecologist and surgical work experience with plastic surgeons****. – Participant 1 SNS*  *I knew that, in your application you needed to umm, include something about being in a role of care. And* ***I was a little bit worried*** *about, whether that was going to be, errm, in the same bracket because it was in a school,* ***it wasn’t in a nursing home, or what normally people do which seems to be a nursing home. -***  *Participant 12 SS*  *I started to volunteer there from the very beginning of year 12 every Tuesday afternoon because* ***I knew that voluntary work would be good until interviews. –*** *Participant 11 SS* |
| Reflections | Confirm career choice | *And that’s probably why I now realise why all my teachers say doing work experience is so important. Because* ***it’ll either tell you it’s what you want to do. Or tell you,” no, this is definitely not what I want to do”*** *– Participant 15 SS*  *I think speaking to doctors was also helpful and work experience because they would sort of encourage you to do it but also make you* ***aware of the negative aspects of becoming a doctor****, especially in their specialty and the reasons why they went into their specialty, maybe what they would do differently, things like that. –Participant 14 SS*    *At first, I thought it was all, I was more like I’m doing this more for* ***my own personal needs, I was like I need it, have it for my application.*** ***But doing it later on, I really enjoyed myself and I felt like that was actually the biggest part of my application*** *– Participant 18 IND* |
|  | Shifting attitudes | *I think before I went to the GP, I thought people went to the GP when they have minor things, so when they have colds, infections, but when I was there, I realised there was a lot of problems to do with mental health and it exposed me to things that.* ***If I hadn’t been in a GP, I wouldn’t have found that out and witnessed it in the way that I did. –*** *Participant 22 IND*  ***He did educate me a lot about the whole process****. And the entrance exams, and what kind of things I should be doing to help like, help me have a better chance of getting an interview. Like he’s the one who advised me that I should be spending time in a care home and I should be spending time at school. – Participant 9 SNS*  *It was a good point* ***from there to know what to do to go into some more work experience******…****I still get in touch with umm, the guy who, err, I did work experience with cos we get on really well. So, he’s umm,* ***helped me out through and, yes and so sometimes he recommends things to do. –*** *Participant 12 SS* |
|  | Variable Quality | *I also did weekly volunteering for year 12 and year 13, just to you know* ***pad out the personal statement*** *as well and get as much experience as possible. – Participant 19 IND*  *And I know in interviews you have to speak a lot about your work experience.* ***So, I felt like I had to take, I had to undertake a placement where I actually could connect*** *and know what I was talking about. And so, I was actually passionate about it. And that’s why* ***I actually spent a lot of time applying for more****. – Participant 9 SNS* |
| Volunteering versus clinical shadowing | | *No, I had also previously been a volunteer at a charity shop for one year, um, a couple of years ago. I wanted a* ***more social and interactive*** *work experience placement as opposed to a clinical one. That was of my own doing. – Participant 23 IND*  *Volunteering helps because it gives you things to talk about and it* ***gives you the skill set****, so I started that as well. – Participant 4 SNS*  *Everything helps itself, the two big things, the weekly work experience, you are only there for a couple of hours, so you don’t see anything hugely interesting and think I have to look that up in the same way as when I was following a doctor for a day. Doing it regularly over a period of time,* ***being comfortable in a ward setting, being used to it, a general understanding of the atmosphere,*** *how to deal with patients that’s best for them. – Participant 21 IND* |
| Applicants’ insight into variability and fairness | | *I’ve just been thinking about how many people, maybe with lower class backgrounds, maybe they don’t have the opportunities that I have to like go to a school where they put on like all these interviews and bring in guests, that sort of thing. Um, how much more difficult it must be for them* ***to find within themselves to reach higher*** *to find all these like medical schools, like all the practice alumni interviews um they’re very expensive whereas my school put one on for free and* ***I feel like I’m privileged*** *to be in that position. But I think that maybe someone, maybe a more disadvantaged um position would they have that same opportunity. Um, so I’ve just been thinking about that, a lot. – Participant 16 SS WP*  *There's a lot of like widening participation programmes, so getting people from lower income families to get into medicine. But at the same time, although that's very good trying to broaden the access to medical school, but its kind of* ***disadvantaging people who kind of people expect people to pay a lot for their course*** *if that makes sense. I don't know if that sounds a bit vague, but like for me, I couldn't get onto (summer schools). So, although that’s good for people getting a chance, people would be disadvantaged because they're only able to get onto (a summer school) without paying loads of money for it. – Participant 10 SNS* |
